# Global network for women’s and children’s health research: a system for low-resource areas to determine probable causes of stillbirth, neonatal, and maternal death

**DOI:** 10.1186/s40748-015-0012-7

**Published:** 2015-05-04

**Authors:** Elizabeth M McClure, Carl L Bose, Ana Garces, Fabian Esamai, Shivaprasad S Goudar, Archana Patel, Elwyn Chomba, Omrana Pasha, Antoinette Tshefu, Bhalchandra S Kodkany, Sarah Saleem, Waldemar A Carlo, Richard J Derman, Patricia L Hibberd, Edward A Liechty, K Michael Hambidge, Nancy F Krebs, Melissa Bauserman, Marion Koso-Thomas, Janet Moore, Dennis D Wallace, Alan H Jobe, Robert L Goldenberg

**Affiliations:** RTI International, Durham, NC USA; University of North Carolina at Chapel Hill, Chapel Hill, NC USA; FANCAP, Guatemala City, Guatemala; Moi University Medical Teaching Hospital, Eldoret, Kenya; KLE University’s JN Medical College, Belgaum, India; Latta Medical Research Foundation, Indira Gandhi Medical School, Nagpur, India; University of Zambia, Lusaka, Zambia; Aga Khan University, Karachi, Pakistan; Kinshasa School of Public Health, Kinshasa, Democratic Republic of the Congo; University of Alabama at Birmingham, Birmingham, AL USA; Christiana Health Care, Newark, DE USA; Massachusetts General Hospital, Boston, MA USA; Indiana University, Indianapolis, IN USA; University of Colorado, Denver, CO USA; UNC Chapel Hill, Chapel Hill, NC USA; Perinatology and Pregnancy Branch, NICHD, Bethesda, MD USA; Cincinnati Children’s Hospital, Cincinnati, OH USA; Columbia University Medical Center, New York, NY USA

**Keywords:** Cause of death classification, Maternal mortality, Stillbirth, Neonatal mortality, Low-income Countries

## Abstract

**Background:**

Determining cause of death is needed to develop strategies to reduce maternal death, stillbirth, and newborn death, especially for low-resource settings where 98% of deaths occur. Most existing classification systems are designed for high income settings where extensive testing is available. Verbal autopsy or audits, developed as an alternative, are time-intensive and not generally feasible for population-based evaluation. Furthermore, because most classification is user-dependent, reliability of classification varies over time and across settings. Thus, we sought to develop classification systems for maternal, fetal and newborn mortality based on minimal data to produce reliable cause-of-death estimates for low-resource settings.

**Results:**

In six low-resource countries (India, Pakistan, Guatemala, DRC, Zambia and Kenya), we evaluated data which are collected routinely at antenatal care and delivery and could be obtained with interview, observation, or basic equipment from the mother, lay-health provider or family to inform causes of death. Using these basic data collected in a standard way, we then developed an algorithm to assign cause of death that could be computer-programmed. Causes of death for maternal (trauma, abortion, hemorrhage, infection and hypertensive disease of pregnancy), stillbirth (birth trauma, congenital anomaly, infection, asphyxia, complications of preterm birth) and neonatal death (congenital anomaly, infection, asphyxia, complications of preterm birth) are based on existing cause of death classifications, and compatible with the World Health Organization International Classification of Disease system.

**Conclusions:**

Our system to assign cause of maternal, fetal and neonatal death uses basic data from family or lay-health providers to assign cause of death by an algorithm to eliminate a source of inconsistency and bias. The major strengths are consistency, transparency, and comparability across time or regions with minimal burden on the healthcare system. This system will be an important contribution to determining cause of death in low-resource settings.

## Background

Maternal, fetal and newborn mortality rates remain high in low-resource settings [1-3]. A medical cause of death is an important first step in strategy development to reduce these deaths and to measure changes in death rates from specific causes [4-7]. To date, more than 35 systems have been developed to classify the cause of stillbirths alone, and other classification schemes attempt to define causes of neonatal and maternal deaths [8-12]. Most of these classification systems are best suited for high income settings because the tests to define cause of death are extensive. Few of the classification systems are targeted at low-resource settings where more than 98% of deaths occur. In many low-income countries, minimal resources are available for determining cause of death for mothers, much less cause of death for fetuses and newborns which occur much more frequently, and diagnostic tools such as autopsy, placental histology, x-ray, ultrasound and bacterial cultures are generally not available [13].

Dependence on detailed diagnostics makes many of the existing classification systems quite complicated. Many also use several different types of constructs to determine cause of death including primary and secondary causes, associated causes, contributing causes, underlying causes, or preventable causes [9-22]. One system for perinatal mortality, for example, attempts to determine a main cause, an underlying cause and contributing factors [17]. While such systems are useful for research or in areas where the resources are available to determine the many contributions to each death, these systems are too complicated for routine use, especially to ascertain cause of death on a population basis in low-resource settings [4]. The resources required to determine cause of death is important since few of the poorest countries routinely collect cause of death information [14].

The actual cause of death for any individual mother, fetus or newborn is rarely known with a great degree of certainty, especially in resource-poor areas. Some classification systems have attempted to categorize the degree of uncertainty about whether a specific condition caused a specific death by creating categories such as probable cause, possible cause or whether the condition was merely associated with that particular death [10]. While such systems might also be useful in high resource areas or in specific research projects, they are likely to be too resource-intense for population-based estimates.

A related issue for classification systems is the percent of deaths classified as of unknown cause. The more certainty required for classification, the greater the proportion of deaths classified as of unknown cause is likely to be. As an example, the percent of stillbirths classified as having an unknown cause varies widely between classification systems. Depending on the classification system [15] and the level of investigation [16], the proportion of unexplained stillbirths has ranged from 15% to more than 70%. Even in high-income countries, with advanced testing and autopsy, a significant proportion of stillbirths are classified as of undetermined cause [9,23].

Other factors important to all classification systems are how the cause of death is determined and who determines the cause of death [23-26]. A major concern with any cause of death classification system is the reliability of the cause of death determination, over time, for the same evaluator(s), and especially for evaluators in different locations, even when the same information is available. When different clinicians determine the cause of death for any specific case, even with the same information available, major differences in the cause of death often occur [25-28]. For example, for a preterm baby with difficulty breathing at birth, the cause of death may be variably classified as prematurity, respiratory distress syndrome (RDS), asphyxia or pneumonia by different classifiers. Similarly, an anencephalic baby who dies in the neonatal period likely dies of the anomaly itself, but also may die from an infection or asphyxia or both. Different classifiers could evaluate these cases and choose very different causes of death. Thus, in most classification systems, the determination of the primary cause of death may not depend only on the case data available but also on idiosyncrasies of the classifiers. For this and other reasons, including lack of specific guidelines about how to classify cause of death, there have been large variations in cause of death by the system and evaluators [28-30]. In LIC different types of health care providers may classify causes of death differently [27]. But because there has been no gold standard for these evaluations, the actual cause of death is often unknown, and which type of provider comes closest to selecting the “true” cause of death is unclear. While physicians have traditionally been viewed as better at determining cause of death than providers with less training, whether this is the best use of physicians’ or other trained providers’ time is a concern in geographic areas with limited health provider availability.

There are two main types of classification systems, multi-causal and single causal [6,30-32]. The multi-causal approach lists all potential causes and contributing factors, with rules to distinguish ‘primary’ vs. ‘underlying’ or ‘contributing causes’. This type of system may be more meaningful where resources are available to conduct extensive testing and perform analyses. Another type of system includes a hierarchy to select one primary cause of death, when multiple factors are identified and possibly causal [32]. While a limitation to selecting one primary cause of death is that important secondary or contributing factors or nuances for individual cases may be lost, choosing one primary cause helps to increase the consistency of results and likely makes the data easier to comprehend and use by policy makers [5,33]. Thus in addition to reproducibility of results, a single cause system should allow for more meaningful comparisons in the mortality rates associated with specific causes over time and across geographic areas.

One mechanism to inform cause of death for low-resource settings is based on verbal autopsy (VA) [27,34-37]. VA systems have generally been used for determining cause of maternal deaths. VA requires lengthy family interviews which are a burden on the health system and thus are not practical to conduct on a population-basis. VA for stillbirth or neonatal deaths is more burdensome because they are more frequent than maternal deaths [27]. Furthermore, VA interviews may produce variability in assignment of a cause of death based on the classification system used and the person who assigns a cause of death [27]. Furthermore, in many VA systems, while the clinical information may be gathered in a consistent manner, with few exceptions, a coder determines cause of death, with the limitations of reproducibility noted above [35]. Finally, the diagnostic accuracy of VA has been weak in some field studies, with limited ability to accurately determine some specific causes of death [34,37].

## Methods

Our objective was to develop reliable classification systems that would assign cause of maternal, fetal and neonatal death using the minimal amount of descriptive data and would not depend upon individual clinicians for the assignment of cause. Our goal is to increase consistency with a low burden on the health system. We elected to use data that are generally available in low-resource settings from the mother, family or caregivers and that require only basic equipment (e.g., a scale for birth weight determination, blood pressure cuff, or thermometer). However, with increasing rates of facility delivery in low-resource settings, we also elected not to ignore hospital-based information, if available (e.g., chest x-ray diagnosis of pneumonia). We sought to create a system to classify the primary causes of death, that was practical to use and consistent for deliveries occurring at home and other community settings as well as for hospital births. The system described below, the “Global Network Probable Cause of Death Classification” for stillbirth, maternal and newborn mortality was developed within the Global Network for Women’s and Children’s Health Research, a multi-country, research network with sites in Sub-Saharan Africa, Asia and Latin America funded by the *Eunice Kennedy Shriver* National Institute of Child Health and Human Development [38,39].

The underlying principle of the Global Network system was to collect basic and simple observational information related to the pregnancy and death. A second principle was that an algorithm would assign cause of death, removing personal choice or bias from the assignment. The algorithm uses a hierarchical classification system to determine one primary cause of death. The specific causes of stillbirth, neonatal and maternal death defined by this classification system are shown in Table 1 with the rationale for the hierarchy of the system; these causes align with ICD-10 first level classifications [31], as well as with major existing classification systems. Table 2 includes the specific definitions of each cause, as adapted for this system. The advantage of this methodology is that the system can determine which condition is most immediately associated with the death in a consistent manner across all cases. Although this system may at times classify cause of death differently than other systems, we viewed this possibility as acceptable because there is no gold standard for classifying cause of death, and our system has the attributes of transparency and reproducibility.

**Table 1:** **Causes of stillbirth, neonatal death and maternal death and their hierarchical position in the Global Network Classification System**

	**Comment**
**Stillbirth**	
Maternal or fetal trauma	Significant maternal trauma especially if the maternal abdomen is involved or there is evidence of fetal trauma takes precedence as a cause of stillbirth over all other potential causes
Major Congenital anomaly	Major anomaly takes presedence as a cause of death over all other conditions except trauma
Maternal infection	Maternal malaria or syphilis or signs of amnionitis
Asphyxia	Based on the maternal or fetal condition noted including obstructed labor, abruption or previa characterized by antepartum bleeding, preeclampsia/eclampsia, fetal distress and cord complications
Complications of preterm labor	There are some early gestational age stillbirths, generally prior to 24 weeks, where the fetus apparently dies because it is unable to tolerate labor. These very preterm babies are usually not macerated since they usually have died close to delivery
Unknown	No other cause identified
**Neonatal death**	
Major Congenital anomaly	Significant congenital anomaly takes precedence as a cause of neonatal death
Sepsis/pneumonia/tetanus	The presence of these conditions take precedence as a cause of death except when an anomaly is present
Asphyxia	Breathing difficulties at birth with maternal condition noted including obstructed labor, bleeding, preeclampsia/eclampsia, fetal distress, cord complications, etc.
Complications of prematurity	Deaths in preterm infants not attributable to other causes. Since it is difficult to differentiate asphyxia from respiratory distress syndrome, we have arbitrarily assigned larger infants with respiratory distress to asphyxia and the smaller or earlier preterm infants to complications of prematurity.
Unknown	No other cause identified
**Maternal Death**	
Significant maternal trauma	Trauma takes precedence as a cause of maternal death
Abortion/miscarriage/medical termination of pregnancy/ectopic pregnancy	If the subject has a history of abortion or is less than 20 weeks, whether she had hemorrhage, sepsis or other conditions, the cause of death is considered an abortion
Infection	If there is no trauma or an abortion, the presence of significant infection takes precedence as a cause of maternal death
Hemorrhage	The most commonly attributed cause of maternal death in most settings
Hypertensive disease of pregnancy	If mother has a seizure, eclampsia is considered the cause of death. If she has only preeclampsia, other causes may take precedence
Thromboembolism	With no other obvious cause and sudden onset of severe respiratory distress and chest pain, the cause of death will be attributed to thromboembolism
Medical condition coincident to pregnancy	If a medical condition such as cancer, cardiac disease, severe anemia, or diabetes is present and there is no other cause of death, the death will be attributed to a medical condition
Unknown	No other cause identified

**Table 2:** **Definitions to classify causes of stillbirth, neonatal and maternal death in the Global Network Classification System**

**Cause of death**	**Definition**
**Stillbirth**	
Maternal or fetal trauma	Any trauma occurring to the mother during pregnancy including an accident, physical assault, or suicide and/or evidence of traumatic stress to the fetus at time of delivery including severe bruising, cephalohematoma, sub-conjunctival hemorrhage, large caput, long bone fracture, etc.)
Major congenital anomaly	Major congenital malformation or anomaly including neural tube defect, abdominal wall defect or other visible defects
Maternal infection	Evidence of maternal infection during pregnancy or delivery including being positive for malaria, syphilis or presence of fever, significant vaginal or fetal odor at delivery
Asphyxia – *may be associated with maternal preeclampsia/eclampsia, obstructed labor, antepartum hemorrhage, fetal distress or cord accidents*	**Preeclampsia/eclampsia** Characterized by hypertension (blood pressure 140/90 mg Hg) and proteinuria occurring after the 20th week of pregnancy. May include symptoms: severe headache, blurred vision, nausea and/or vomiting, abdominal pain and a diminished urinary output. Eclampsia is characterized by convulsions and coma and may be preceded by signs of pre-eclampsia or the onset may be rapid and sudden.
	**Obstructed/prolonged labor** Descent is arrested during labor due to an insurmountable barrier, despite strong uterine contractions and further progress cannot be made without assistance. Prolonged labor includes labor > one day.
	**Heavy bleeding before delivery** Blood loss of >1000 cc (>4 cups) prior to delivery
	**Signs of fetal distress during labor** Includes decreased fetal movements, fetal bradycardia (<120 beats per minute), fetal tachycardia (>160 beats per minute), and/or meconium stained liquor
	**Cord complication** Includes cord prolapse, cord around the neck, cord compression or cord rupture prior to delivery
Complications of preterm labor	Gestational age <32 weeks or birth weight <1500 g with evidence that the fetus died in labor or was not macerated
**Neonatal death**	
Major congenital anomaly	Includes major anomalies such as neural tube defect or anencephaly, abdominal wall defect, etc.
Infection	Signs include high temperature (fever; very warm to touch or >37.5C) or a very low temperature (cool to the touch or <35.5C); fits/seizures ≥2 days after birth; cloudy discharge, pus or bleeding at the umbilical stump; and for pneumonia, chest x-ray or clinical signs including poor feeding and irritability, as well as tachypnea, retractions, grunting, and hypoxemia.
Asphyxia	In term infants and preterm infants >2000 g: Breathing difficulties at birth, fits or seizures <2 days of birth; Infant received bag and mask or other resuscitation effort at birth; maternal complications associated with neonatal asphyxia including maternal preeclampsia/eclampsia, obstructed labor, breech presentation, twins and antepartum hemorrhage, and fetal distress and cord accidents. (see stillbirth causes for definitions)
Complications of prematurity	All deaths <34 weeks or <2000 gs not due to a congenital anomaly or infection are categorized as due to a complication of prematurity as are deaths in larger or late preterm infants not due to congenital anomaly, infection or asphyxia. Many of the deaths categorized as due to complications of prematurity are due to respiratory distress syndrome. These babies may require resuscitation at birth or develop breathing difficulties within hours of birth
**Maternal death**	
Trauma	Any trauma occurring to the mother during pregnancy including an accident, physical assault, or suicide
Abortion/miscarriage/ectopic pregnancy	Includes any spontaneous or induced pregnancy loss or death of fetus prior to 20 weeks gestation including ectopic pregnancy, defined as Implantation of an embryo somewhere other than the uterus, such as in one of the fallopian tubes
Eclampsia	One or more convulsion or state of unresponsiveness usually associate with hypertension and proteinuria
Hemorrhage	Heavy bleeding with a blood loss of >1000 cc or 4 cups before or after delivery and may be associated with any surgical procedure to stop maternal bleeding
Infection	Evidence of maternal infection during pregnancy or delivery including evidence of malaria, syphilis or presence of significant vaginal or fetal odor at delivery. Evidence of infection includes fever, defined as body temperature higher than normal limit or being very warm to the touch and chills defined as uncontrolled shivering
Preeclampsia	Blood pressure >140/90 mm Hg and proteinuria, headache, and may include stroke, loss of consciousness or paralysis
Thromboembolism	Acute shortness of breath and chest pain which may be associated with prolonged bed rest and lower limb venous thrombosis or clots
Medical conditions	If no other cause is defined, any medical condition such as severe anemia, diabetes, renal disease, etc.

The classification system was designed as part of the Global Network’s Maternal and Newborn Health Registry study, a population-based registry of pregnancy which obtains outcomes from consenting women through 6-weeks postpartum [38]. The institutional review boards and ethics committee at the participating study sites (Aga Khan University, Karachi, Pakistan; Kinshasa School of Public Health, Kinshasa, DRC; Moi University, Eldoret, Kenya; San Carlos University, Guatemala City, Guatemala; University of Zambia, Lusaka, Zambia) and their affiliated U.S. partner institutions (University of Alabama at Birmingham, University of North Carolina at Chapel Hill, Columbia University, University of Indiana, Christiana Healthcare, and Massachusetts General Hospital) and the data coordinating center (RTI International) approved the study.

## Results and discussion

### The stillbirth classification algorithm

Stillbirths are generally considered to be deaths in utero occurring at 20 weeks gestation or greater, depending on the setting [40]. Among maternal, fetal and neonatal deaths, determining cause of stillbirth has historically been the most challenging type of death to define, as the fetus is not directly observed when death occurs [6]. To date, cause of death in stillbirths has generally been determined from the underlying maternal or obstetric conditions that may be directly or indirectly associated with the fetal death. Additionally, autopsy and placental data may be used to help classify of cause death in stillbirths in high resource settings. At least one high-income country system primarily attributes the cause of stillbirth to placental causes [16], and placental conditions are considered in many other stillbirth classification systems [41]. However, despite their value in determining cause of death in high-income settings, we have deliberately chosen not to include autopsy and placental findings in this classification system since autopsies are almost never done and placentas are rarely examined histologically in low-income settings.

Where antenatal care is limited and a significant proportion of deliveries occur in home or low-level clinics with community birth attendants [42], distinguishing stillbirth from early neonatal death has been problematic [43]. Thus, some authors have proposed a classification system in which ‘intrapartum death’ encompasses both stillbirths and early neonatal deaths due to intrapartum causes such as asphyxia [44]. To date, no system to determine cause of stillbirth with basic data has been widely used [6]. To address these issues with an emphasis on low-resource settings, our system first distinguishes stillbirth from miscarriage/abortion through utilizing the lower limit of 20 weeks gestation (or 500 g if GA is unavailable). We next distinguish stillbirth from neonatal death by whether any signs of life such as a heartbeat, crying, breathing or movement are present at delivery. Because distinguishing antepartum deaths from intrapartum deaths may be crucial for designing interventions in the appropriate time period, the system also considers whether signs of maceration are present, suggesting that the stillbirth likely occurred >12 hours prior to the delivery and was likely antepartum [45].

In low resource settings, for most stillbirths, whether antepartum or intrapartum, the final common pathway is most likely asphyxia. However, even with placental and autopsy data, it is difficult to prove that a fetus died of asphyxia. Thus we have chosen to focus on the presence of maternal and fetal conditions (e.g. abruption, preeclampsia) highly predictive of asphyxia. Therefore, signs and symptoms addressing maternal and fetal conditions that have been associated with stillbirth are also specified. These include obstructed labor, antepartum or intrapartum hemorrhage, preeclampsia/eclampsia, cord complications, fetal distress and intrauterine growth restriction.

**Figure 1:**
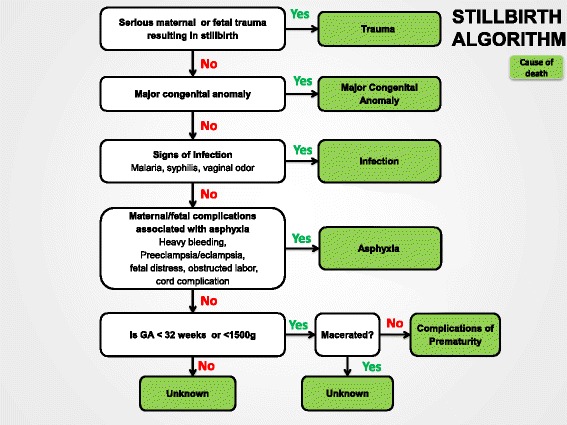
**Algorithm to classify causes of stillbirth.**

The criteria for assigning a cause of stillbirth are shown in Table 2. Our hierarchical method of determining cause of stillbirth relies on a discreet data set. The algorithm first determines whether the stillbirth was associated with maternal or fetal trauma (i.e., assault, suicide, accident, fetal trauma); if so, the cause of death is classified by algorithm as trauma. Second, if trauma did not occur and there is a major (visible) congenital anomaly, the death is categorized as due to a congenital anomaly. If neither trauma nor anomalies are identified and signs of maternal or fetal infection are present, the stillbirth is classified as due to infection. If none of the above are present and any of a list of maternal or fetal conditions associated with intrauterine asphyxia are present, the cause of death is classified as asphyxia. (The specific maternal or fetal condition likely leading to the asphyxia is noted.) In many areas, very preterm fetuses in labor even with distress are not delivered by cesarean section because they do not survive in the neonatal period and are allowed to die in labor. We therefore have created a category of stillbirth due to preterm labor to capture these stillbirths. If the stillbirth does not fit into one of these categories, only then is it classified by algorithm as of unknown cause. Thus, using these categories, the stillbirth cause of death is classified by the major conditions associated with the fetal death (Figure 1).

### Neonatal death

Neonatal deaths are defined as live births with a death occurring at less than 28 days. The main conditions associated with neonatal death in low-resource areas are asphyxia, sepsis/infection, and complications of preterm birth, with major congenital anomalies less commonly a cause (by percentage) in low compared to high and middle-income countries.

There are many difficulties in assigning cause of death in neonates even in high-income countries with x-ray, culture and autopsy availability. For example differentiating sepsis and asphyxia is difficult even in term births, while in preterm births where respiratory distress syndrome is a common cause of respiratory failure and death, distinguishing among these three causes of neonatal death is even more difficult.

**Figure 2:**
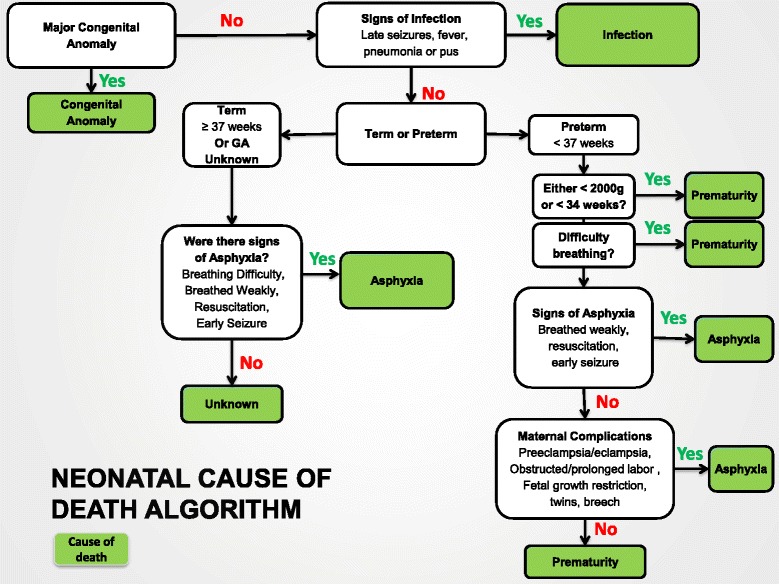
**Algorithm to classify causes of neonatal death.**

The criteria for assigning a cause of neonatal death are shown in Table 2. In our system, we first determine if a major congenital anomaly is present (Figure 2). If so, the algorithm assigns congenital anomaly as cause of death. If an anomaly is not present and signs of infection are present (e.g., tetanus, omphalitis, sepsis, pneumonia (signs such as late onset respiratory difficulty, fever or hypothermia or X-ray if available), infection is assigned as the cause of death. If neither an anomaly nor infection is present, the algorithm then separates the deaths into those occurring in term or preterm infants. In the term infants, if there were signs of breathing difficulty or no cry at birth, the algorithm assigns the cause of death as birth asphyxia. The maternal or fetal condition likely associated with the birth asphyxia is noted. If no signs of difficulty breathing at birth or respiratory distress were present, the cause of death is assigned as unknown.

For preterm infants, especially those ≥2000 grams or ≥34 weeks at birth, among those with breathing difficulties or no cry at birth, asphyxia is a common cause of death [46]. In those infants, if breathing difficulty or no cry is present at birth, and maternal conditions such as abruption associated with asphyxia are present, the algorithm assigns cause of death as asphyxia. Otherwise, the cause of death is assigned to prematurity. If the infant is <2000 grams or <34 weeks at birth, the algorithm assigns the cause of death as being due to preterm birth regardless of whether respiratory distress is present, since RDS is common and pneumonia has previously been considered and rejected as a cause. In infants born at <37 weeks, with no congenital anomaly or infection, the algorithm does not classify any death as of unknown cause, because prematurity is always considered as the primary contributor to death.

### Maternal death

Maternal deaths generally are defined as those that occur at any time during pregnancy up to 6 weeks post-partum, regardless of the cause. Maternal deaths are rare compared to stillbirths and neonatal deaths, and fewer classification systems exist to assign cause of maternal death. Furthermore, compared to neonatal deaths or stillbirths, maternal deaths are less likely to have an ‘unknown’ cause of death. However, some reports suggest misclassification of maternal deaths (i.e., not recognizing a woman was pregnant at time of her death), with under-reporting of maternal mortality. Maternal deaths have generally been classified as directly or indirectly associated with pregnancy (e.g. medical causes not brought on or exacerbated by the pregnancy or trauma) [47]. Obstetric conditions directly associated with maternal death include hypertensive diseases of pregnancy (preeclampsia/eclampsia), obstetric hemorrhage (ante- or postpartum, with or without severe anemia), sepsis/infection and thromboembolism. Obstructed labor may be associated with maternal death, leading to either hemorrhage or severe infection but the primary cause of death in the current World Health Organization (WHO) international classification system (ICD-10) would be infection or hemorrhage, not obstructed labor. Deaths associated with ruptured uterus are presumed to be secondary to hemorrhage. Conversely, abortion related deaths result from infection or hemorrhage, but deaths occurring at less than 20 weeks gestational age, including from ectopic pregnancy, are classified as abortion related. Indirect causes of maternal death include trauma or medical conditions such as cardiac disease, cancer, or diabetes.

**Figure 3:**
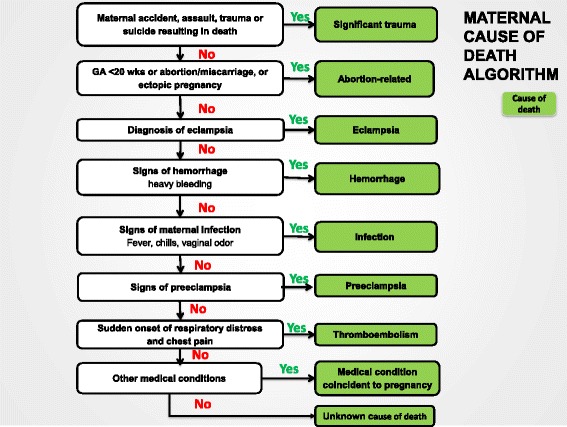
**Algorithm to classify causes of maternal mortality.**

In the Global Network classification system, to assign a cause of maternal death, the major clinical signs and symptoms most often associated with maternal death are identified and defined (Table 2). Next, we developed an algorithm to assign cause of death based on the clinical signs (Figure 3). The algorithm first identifies significant maternal trauma and if present, the cause of death is trauma. If there is no trauma and the pregnancy is less than 20 weeks or an abortion was induced at ≥20 weeks, the cause of maternal death is classified as abortion related. If neither of these is present, and the woman experienced a seizure, eclampsia is considered the cause of death. If none of these are present and any signs of hemorrhage are present, hemorrhage is assigned as the cause of death. If none of the above are present and signs of infection are present, infection is assigned as the cause of death. Next, other signs of hypertensive disease and especially preeclampsia are handled in a similar manner. If at this point, acute shortness of breath and chest pain are present, thromboembolism would be considered the cause of death. Finally, if none of the above are present, the algorithm considers medical conditions not directly associated with the pregnancy, such as renal disease, heart disease, cancer or diabetes, and if any of these are present, the medical condition is assigned as the cause of death. If none of the above is present, the cause of death is classified as unknown.

## Conclusions

The Global Network classification system uses minimal, basic data from the mother, family or lay-health providers. No laboratory tests, placental examinations or autopsies are necessary. Easily identifiable signs are noted and collected in a standard way and entered into a database. The cause of death is then assigned by an algorithm. No person assigns the actual cause of death which eliminates a source of inconsistency and bias. Thus the major strengths are consistency and transparency, with an ability to provide comparability across time or regions with minimal burden on the healthcare system. Even if one does not completely agree with the algorithm, the method of assignment is transparent. Also, since all data used to inform the cause of death assignment reside in the database, alterations and/or improvements in the algorithm at a later time will permit reclassification of the cause of death.

The system assigns a single cause of death, although, the algorithm could be altered to select several possible causes if that output is desired. For example, using this system in addition to the primary cause, other conditions that also were present as secondary conditions could be characterized without relying on the subjective judgment of researchers or caregivers. Additionally, other clinical or laboratory questions that might better help to assign cause of death could be added, depending on available resources and the setting where the death occurred. For now, however, we believe that the assignment of a single cause of death is sufficient to guide most public health and medical system policy decisions. More nuanced assignment of cause of death, such as identification of the type of infection that caused a death, would require additional data and is beyond the scope of this system. The system uses the major causes of death that have been well-established, and are commonly used for cause of death classifications, especially in low-resource settings. These attributes make this system potentially useful both for research and public health policy purposes.

We recognize that this system necessarily is a simplification compared to more complicated systems, and subtle and rare causes of death in low-income settings may be missed. It also does not attempt to address social or other factors that may contribute in low-resource settings. Preventable causes of death are not specifically addressed as such. However, with these limitations, the major causes of death related to pregnancy are collected and the portion of deaths attributable to the major causes can be quantified.

We have developed a system to classify causes of death for stillbirth, neonatal and maternal death that should be applicable for low-resource settings. In these areas, where most pregnancy-related mortality occurs, reliable and reproducible classification of maternal, fetal and neonatal death is needed both to advance research and to inform public health strategies to reduce pregnancy-related mortality. While preliminary analyses have been done to address the system, validation of the system is ultimately necessary, and this system should be compared to other classification systems. A reliable system to determine cause of death will ultimately serve to inform public health strategies necessary to reduce the high maternal, fetal and newborn mortality burden in low-resource settings.
